# Remarkable Case of Strangulated Hernia, Successfully Treated

**Published:** 1848-05

**Authors:** J. Dunott


*Remarkable Case of Strangulated Hernia, successfully treated.*
By J. Dunott, M. D.

John Conler, aged thirty-four years, has been the subject of Scrotal Hernia of the right side from infancy.

He recollects having worn a truss when very young, but had abandoned its use for many years, and experienced no inconvenience, except an occasional protrusion of the bowels, when he made some great or unusual bodily exertion, to which his ordinary occupation as a labourer now and then subjected him. He was always able to replace the gut and return directly to his work.

About fourteen years ago, he procured a truss at the instigation of a physician in Boston, which he has worn ever since, with some trifling intervals, and thinks he has not been able to return the gut with the same facility since.

My attention was called to him on the 16th of January, by his attending physicians, Drs. Smiley and Ashton. I found him with frequent and somewhat resistant pulse, abdomen tympanitic and tender, frequent vomitings of dark but not offensive matter, countenance pale and indicative of much distress. The right side of the scrotum very much distended, measuring from twelve to fourteen inches in circumference, and exceedingly tense.

These symptoms had resisted all usual remedies in such cases for forty-eight hours. A liberal dose of castor oil, with the addition of a drachm of spirits of turpentine, was now given, and followed in two hours by an enema, which procured three pretty copious evacuations; after this an opiate was administered, and an effort made to restore the parts by taxis, aided by local applications. By these means the tumour was considerably reduced in size, and the more urgent symptoms abated. He passed a tolerable night, but on the following morning his stomach became exceedingly irritable, rejecting everything, and in the after part of the day vomiting almost constant and stercoraceous; no evacuation from the bowels; abdomen tympanitic and tender; countenance pale and anxious; pulse quick and feeble; tumour increasing and tender.

It was now thought advisable to divide the stricture without further delay. I accordingly made an incision four inches in length, commencing three-fourths of an inch above Poupart's ligament; and after dividing the faciæ, opened the sac. Nearly a a pint of bloody serum issued from the opening, and the protruded portions of the intestine, consisting of five or six inches of the colon; the cœcum, vermiform appendage, which was doubled upon itself, adherent and indurated, and four or five inches of the ilium, all of a dark mahogany colour, were exposed.

The stricture being divided, a slight effort was made to bring down the intestine with a view to examine the strictured portion. This was found to be impracticable in consequence of the close connection of the intestines to the inguinal ligaments and ramus of the pubis, and on passing the finger behind them it could not be carried higher than the inferior edge of Poupart's ligament. On raising the intestine, the meso-colon, which had been brought to this position by the descent of the peritoneum, was seen to be continuous with the posterior portion of the sac. This condition of things rendered the replacing of the gut an exceedingly difficult task.

After endeavouring to accomplish this end for nearly an hour, with but little effect, I began to be apprehensive it could not be attained. My friend, Professor Pancoast, was called in at this period, and by continuing the effort for nearly an hour longer, with his assistance we were at last successful.

The patient is now (Feb. 22d) at his usual employment, having recovered without any unfavourable symptoms. I have thought the above case one of sufficient interest for publication, because of its novelty and the difficulties necessarily incidental to it.

It is rare to find the cœcum in a hernial protrusion, for it is so closely connected with the posterior parietes of the abdomen, (having little or no mesentery,) that it cannot descend unless the peritoneum be dragged down so as to bring its connections in close proximity to the ring, in which case its laminæ will be separated in such a manner as to do away with it almost entirely, and the gut will be found so closely connected with the inguinal ligaments and parts contiguous thereto, as to render its return almost impossible, and the amount of handling necessary to replace it will greatly augment the danger of the operation.

The result of this case has shown, however, that these may be encountered, and is, perhaps, not the least interesting feature presented by the case.

*Feb. 26th*, 1848.

---

PHILADELPHIA, APRIL 1st, 1848.

To the Editor of the Medical Examiner.

*Dear Sir*,—As promised at a post period, I herewith hand you for publication, a method for the stoppage of maxillary hæmorrhage, that I have been in the habit of resorting to for twenty years past, and which has never failed to accomplish the desired purpose, in a single instance, in the course of eight or ten minutes.

I take common sulphate of copper, broken into small pieces, (but *not* powdered,) and envelope it in a pledget of common cotton, sufficiently thick to hold the same in the exact form the case may seem to require, and when pressed to its proper place, confine it by a compress, or otherwise as circumstances may call for. By the adoption of this simple mode, I have had no case where (the compress being in place) on the third rince of the mouth, the water was not ejected in a perfectly clear and transparent state.

It is well known that sulphate of copper has been used from time immemorial for the stoppage of hemorrhage; but in many cases it has failed, as well as all other known styptics, to accomplish the purpose, when used in the ordinary way. I have supposed, from the time of my first discovering the singular efficacy of this mode of application, that the fibre of the cotton contributed, somewhat, to the styptic power of the sulphate in aid of coagulation, &c.: but since the discovery of "gun cotton," so called, and particularly since the discovery of Mr. S. L. Bigelow, of the Tremont Medical School of Boston, as read before the Medical Society by Dr. J. H. Bigelow, I have been fully convinced I was right in my conjecture.

